# Magnetic Nanoparticle-Assisted Tunable Optical Patterns from Spherical Cholesteric Liquid Crystal Bragg Reflectors

**DOI:** 10.3390/nano7110376

**Published:** 2017-11-08

**Authors:** Yali Lin, Yujie Yang, Yuwei Shan, Lingli Gong, Jingzhi Chen, Sensen Li, Lujian Chen

**Affiliations:** 1 Department of Electronic Engineering, Xiamen University, Xiamen 361005, China; phoebe0327@stu.xmu.edu.cn (Y.L.); 23120171152983@stu.xmu.edu.cn (Y.Y.); 23120171152948@stu.xmu.edu.cn (Y.S.); 23120141153096@stu.xmu.edu.cn (L.G.); 22920142203654@stu.xmu.edu.cn (J.C.); sensenli@xmu.edu.cn (S.L.); 2 Shenzhen Research Institute of Xiamen University, Shenzhen 518057, China

**Keywords:** magnetic nanoparticles, cholesteric liquid crystal, Bragg reflection, microfluidics

## Abstract

Cholesteric liquid crystals (CLCs) exhibit selective Bragg reflections of circularly polarized (CP) light owing to their spontaneous self-assembly abilities into periodic helical structures. Photonic cross-communication patterns could be generated toward potential security applications by spherical cholesteric liquid crystal (CLC) structures. To endow these optical patterns with tunability, we fabricated spherical CLC Bragg reflectors in the shape of microshells by glass-capillary microfluidics. Water-soluble magnetofluid with Fe_3_O_4_ nanoparticles incorporated in the inner aqueous core of CLC shells is responsible for the non-invasive transportable capability. With the aid of an external magnetic field, the reflection interactions between neighboring microshells and microdroplets were identified by varying the mutual distance in a group of magnetically transportable and unmovable spherical CLC structures. The temperature-dependent optical reflection patterns were investigated in close-packed hexagonal arrangements of seven CLC microdroplets and microshells with inverse helicity handedness. Moreover, we demonstrated that the magnetic field-assisted assembly of microshells array into geometric figures of uppercase English letters “L” and “C” was successfully achieved. We hope that these findings can provide good application prospects for security pattern designs.

## 1. Introduction

The concurrent existence of order and mobility renders liquid crystals (LCs) a unique class of soft functional materials for advanced photonic applications [1,2,3]. Fortunately, although cholesteric liquid crystals (CLCs) probably could not attract considerable attentions from the industrial community of liquid crystal (LC) displays due to their disadvantages such as slow response time and high driving voltage, they have still enriched the fundamental knowledge of helical superstructures induced by self-assembly and found innovative (non-display) applications based on selective Bragg reflection of circularly polarized (CP) light [4]. Today, there is a burgeoning interest in the use of LCs with unusual aplanar geometries [5,6,7,8]. In particular, spherical CLC microstructures with a radial orientation of the helical axes, such as microdroplets and microshells, were investigated as Bragg resonators to construct omnidirectional tunable microlasers operating in the pronounced whispering gallery (WG) mode and distributed feedback (DFB) mode [9,10,11,12,13,14].

Benefiting from the rapid evolution of microfluidic technologies, the size-polydispersity problem of spherical LC microstructures was successfully overcome, thus paving an attractive way to fabricate sufficiently monodispersed emulsions with controllable geometrical parameters. Recently, photonic cross-communication, arising from light reflections of different wavelengths and handedness orientations in all directions between well-defined spherical CLC microstructures, has generated dynamically tunable multicolored patterns with a specific spatial distribution and has shown potential for chiroptical all-optical distributor/switch and countless security applications [15,16,17,18]. The employ of photoresponsive molecular switches enabled a wide tuning range of the pitch length of CLCs and hence of the highly selective CP reflection wavelength emanating from paired spherical CLC Bragg reflectors [16]. In addition, it was reported that the transition from droplets to shells gave rise to sharp patterns and sustained excellent optical quality even after polymerization [19,20].

The arrangement of spherical CLC Bragg reflectors is critical for generating specific patterns. However, to date, the underlying assembly methods to achieve ordered arrays are quite limited, usually induced by flow, gravity, or evaporation, etc. In general, the distance between two nearby droplets/shells cannot be altered arbitrarily and the obtained close-packed hexagonal superstructures usually consist of numerous droplets/shells. Also, it is difficult to manipulate each individual spherical CLC Bragg reflector and organize relatively small amounts of them into various separated geometric figures independently. Very recently, by means of magnetic manipulation strategies, the noncontact transport of CLC microshells was successfully achieved with microfluidic devices [13,19]. Chen et al. fabricated dye-doped CLC microshells encapsulated with water-dispersible Fe_3_O_4_ nanoparticles for a magnetically-transportable tunable microlaser [13]. Park et al. also demonstrated the capability of solidified CLC microshells with Fe_3_O_4_ nanoparticles as new types of location-adjustable sensors for the detection of temperature changes, solvent quality, and humidity [19].

Here, we report the microfluidic fabrication of spherical CLC Bragg reflectors in the shape of microshells for magnetic field-assisted optical patterns via photonic cross-communication. Water-soluble magnetofluid consisting of magnetic Fe_3_O_4_ nanoparticles was selectively incorporated in the inner aqueous core of two types of CLC shells, responsible for the non-invasive transportable capability. We investigated the reflection interaction between neighboring spherical CLC Bragg reflectors that were identified by varying the distance in a group of microshells encapsulated with and without Fe_3_O_4_ nanoparticles. With the aid of the non-contact control under a magnetic field, CLC droplets and shells with inverse helicity handedness were closely packed into hexagonal arrays. The temperature-dependent tunability of optical reflection patterns were discussed in detail. Moreover, we successfully achieved the magnetic field-assisted assembly of CLC microshells into the geometric figures of uppercase English letters “L” and “C”.

## 2. Materials and Methods

### 2.1. Materials

Two types of CLC mixtures with inverse helicity handedness were used in the experiment. Mixture I was prepared by adding 2.22 wt % right-handed (RH) chiral dopant R5011 (HCCH) into 97.78 wt % achiral nematic LC E7 (Xianhua, Yantai, China), resulting in the photonic bandgap (PBG) of the CLC locating in the visible light region with the central wavelength around 635 nm. Mixture II was prepared by adding 27 wt % temperature-responsive left-handed (LH) chiral dopant S811 (Xianhua) into 73 wt % E7. The central wavelength of mixture II was about 690 nm at 27 °C and it underwent a blueshift as the temperature increased. The two CLC mixtures were heated above clear point in an oven and mixed ultrasonically until uniform.

Deionized (DI) water dissolved with 10 wt % polyvinyl alcohol (PVA, molecular weight (MW) = 70,000–80,000, 85% hydrolyzed, Aladdin reagent, Shanghai, China) was used as the aqueous phase to enforce planar degenerate anchoring on both inner and outer boundaries, meaning that the LC molecules are forced to lie tangentially near the interfaces. A small proportion of 5 wt % magnetic fluid EMG605 (Ferrotec) consisting of hydrophilic Fe_3_O_4_ nanoparticles was then homogeneously mixed with the PVA solution to render the microshells magnetically transportable.

### 2.2. Fabrication of Cholesteric Liquid Crystal Shells/Droplets

**Figure 1:**
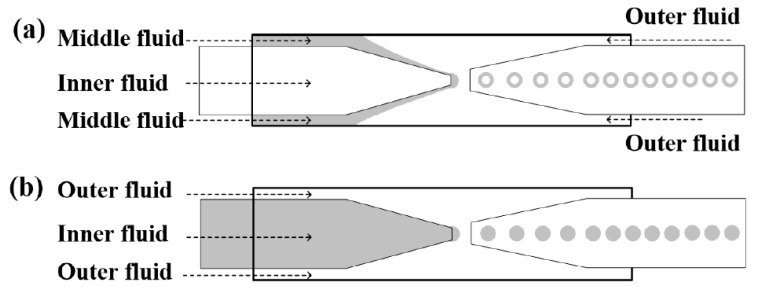
Schematic diagrams of glass capillary microfluidic setups for producing (**a**) water-in-oil-in-water (W/O/W) double emulsion microshells and (**b**) oil-in-water (O/W) microdroplets.

Two kinds of glass capillary microfluidic devices were used to fabricate spherical CLC shells and droplets [21], as shown in Figure 1. Figure 1a was used to fabricate monodisperse microshells as a water-in-oil-in-water (W/O/W) double emulsion. By using the EMG605 and PVA solution as the inner phase, we obtained magnetically transportable microshells (hereinafter, *M*-shells). By using PVA solution as the inner phase, we obtained microshells without magnetic transportability (*S*-shells). In both of these two samples, CLC mixture I served as the middle oil phase and PVA solution as the outer aqueous phase. The device in Figure 1b was used to fabricate microdroplets (*T*-droplets) with CLC mixture II as the inner oil phase and PVA solution as the outer aqueous phase. The samples were collected, selected, and sealed in rectangle glass capillaries for further optical observation.

### 2.3. Optical Characterization

A cross-polarized optical microscope (POM, PM6000, Jiangnan Novel Optics, Nanjing, China) equipped with a charge coupled device (CCD) camera (DCC1645C, Thorlabs, Newton, NJ, USA) was used to measure the size and thickness of microshells and to observe the cross-communication. The numerical aperture (NA) of the objective was 0.25, which means that light with an incident angle smaller than 29° could be collected and measured.

Mixtures I and II were separately filled into planar alignment cells and their reflection spectra were measured at various temperatures. A heating stage (THMS 600, Linkam, Surrey, UK) was used to control the temperature of the samples. A fiber spectrometer (USB4000, Ocean Optics, Shanghai, China) connected to a computer was used to collect the spectra.

## 3. Experimental Results and Discussions

### 3.1. Magnetic Movement of CLC Microshells toward Distance-Dependent Reflections

**Figure 2:**
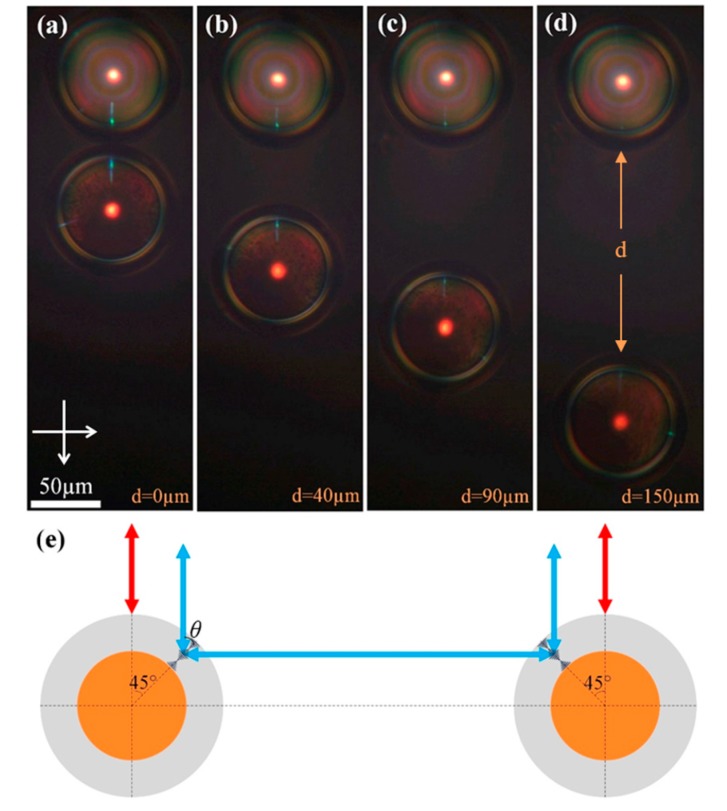
(**a**–**d**) Polarized optical microscope (POM) images of the cross-communication phenomenon with distance-dependent intensity. The cross arrow is the mutual position of crossed polarizer. (**e**) Schematic mechanism of the lateral reflection between two microshells.

Experimentally, the disclinations in cholesteric droplets and shells were not identified in the reflection mode. So, we suppose that the influence of disclinations on the observation of cross-communication arising from light reflections is weak. In addition, the physical contact between neighboring droplets and shells is avoided by PVA, acting as a surfactant to stabilize the emulsion and preventing the droplets/shells from coalescence and collapse. The phenomenon of the intensity of cross-communication spots between CLC droplets becoming dimmer as their mutual distance increases has been reported previously [16]. One of the main shortcomings in obtaining such intensity-variable optical patterns with cross-communication spots is the randomly-packed structures, since the movement of droplets and their mutual distance cannot be precisely controlled as designed. Nowadays, the separated CLC microshells encapsulated by magnetic nanoparticles are endowed with the ability to be transported, positioned, and gathered together by a magnet. In this experiment, the Fe_3_O_4_ nanoparticles dispersed in the inner aqueous phase were chemically modified to be hydrophilic and stay in the core owning to the oil-water immiscibility. They were unlikely to immigrate into oil CLC phase and accumulate in the disclinations in the shell [13,19]. This situation is different to that found in lyotropic spherical CLC structures [7,8]. *M*-shells and *S*-shells with the same diameter of ~100 μm and thickness of ~15 μm were chosen to study the dependence of the cross-communication effect on their mutual distance. From a technical point of view, within a short time of the magnetic manipulating process, the *S*-shells without Fe_3_O_4_ nanoparticles cannot be repositioned by thermal agitation of the outer fluid. Figure 2a–d show the POM images of this process. The upper microshell with a brighter core is the unmovable *S*-shells, while the lower microshell with a darker core is the magnetically transportable *M*-shells with Fe_3_O_4_ nanoparticles. It was found that the intensity of the reflection spots ascribed to the cross-communication between them became weaker and almost vanished when their distance reached more than 150 μm, as the *M*-shells were moved stepwise away from the *S*-shells. Notably, there are some blurry colored circles in *S*-shells which may possibly be contributed by the internal reflections from the interface between the inner aqueous core and the CLC shell. As for the *M*-shells, the inner core looks much darker because of the light scattering effect inside the CLC shell with the presence of magnetic nanoparticles that are dispersed in the aqueous core. 

Figure 2e is the schematic illustration of the involved mechanism of lateral communication between two microshells with the same pitch and the same helicity handedness. Actually, the density of the aqueous core is lower than that of the LC shell, leading to a potential asymmetric geometry by the interplay between buoyancy and gravity. Meanwhile, a symmetric shell geometry is anticipated to be formed due to the elasticity of the cholesteric helix. Taking all the aforementioned elements into account, we assume that the newly fabricated microshells keep symmetric structures for a long time during the microscopic characterization. The liquid crystal molecules at both inner and outer surfaces of the microshells are planar anchored, resulting in the radial orientation of the helical axes. The incident and reflected lights follow the Bragg condition equation *λ* = *np*cos*θ*, where *λ* is the wavelength of the incident and reflected lights, *n* stands for the average refractive index of the CLC, *p* is the pitch of the CLC, and *θ* is the incident angle indicated in Figure 2e. When *θ* = 0°, *λ* is calculated to be ~635 nm, which means that the central red spot corresponds to the selective reflection of normal incidence. For *θ* = 45°, the light would reflected to the horizontal direction, enter the contiguous microshells, and reflect again in the vertical direction with the wavelength *λ* of ~450 nm. The observed red and blue colors corresponding to the central and lateral reflection spots are in accordance with the calculations, respectively.

### 3.2. Influence of Handedness and Pitch on Tunable Optical Patterns in Close-Packed Hexagonal Arrays with CLC Microdroplets and Microshells by a Magnet

**Figure 3:**
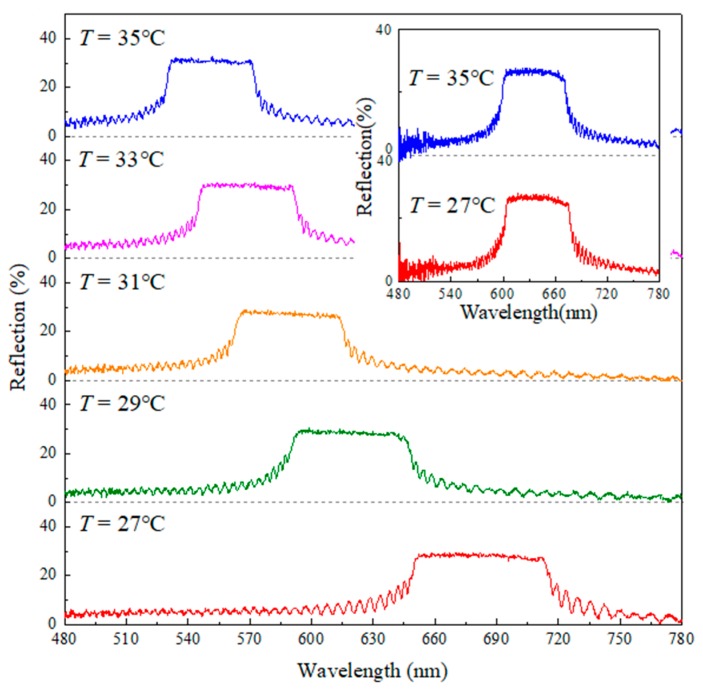
The reflection spectra of thermosensitive cholesteric liquid crystal (CLC) mixture II measured from 27 to 35 °C. The inset shows the thermostability of the reflection band of CLC mixture I in the temperature range studied.

CLCs can spontaneously form into photonic band structures with periodic dielectric helical arrangements. The anisotropic nature of the LC molecules, combined with the continually rotating director *n*, results in the existence of a reflection band for CP light with the same rotation sense as the helix. The co-handed CP reflection is said to be highly sensitive and can only be realized for a small incident angle [22,23]. We chose two CLCs with inverse helicity handedness and different thermal sensitivities to study the tunable optical patterns induced by cross-communication. Since *T*-droplets doped with the LH chiral molecule S811 possess significant thermosensitivity and *M*-shells doped with the RH chiral molecule R5011 are far less sensitive to temperature, we could also easily vary the temperature to examine the reflection of different pitch combinations. As depicted in Figure 3, the wavelength reflection center of mixture II shifts to the blue side from 690 to 570 nm by changing the temperature from 27 to 35 °C. In addition, the inset in Figure 3 confirms the thermo-stable reflection band of mixture I in the temperature range studied.

**Figure 4:**
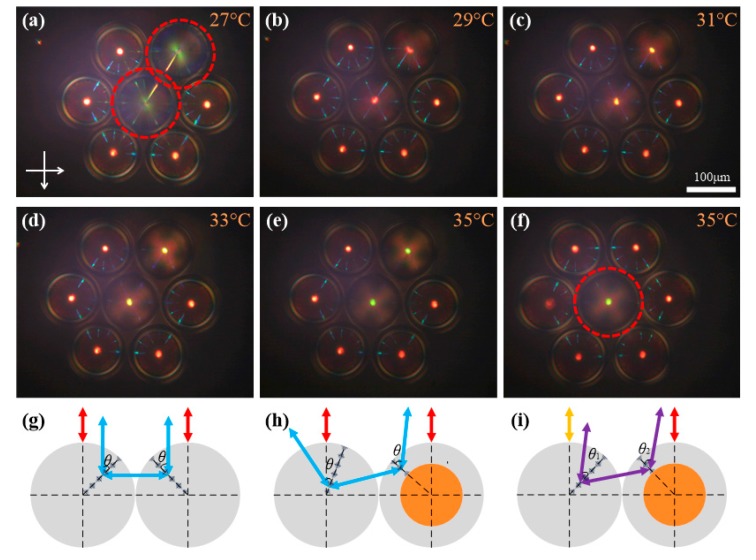
(**a**–**f**) POM images of the cross-communication between microdroplets and microshells with different pitches and inverse helicity handedness in close-packed hexagonal arrangements. The samples circled in red are *T*-droplets. (**g**–**i**) Schematic mechanism of the reflection between (**g**) microdroplets, (**h**) microdroplets and microshells with same pitch, and (**i**) microdroplets and microshells with different pitches.

Figure 4a–f show the cross-communications among several *T*-droplets and *M*-shells in close-packed hexagonal arrangements. To make a close-packed hexagonal arrangement, we tried different proportions of these two samples and found that five *M*-shells together with two *T*-droplets or six *M*-shells with one *T*-droplet could form better arrangements. Although the *T*-droplets are unable to be moved by a magnet, they can still be driven by the neighboring *M*-shells. The arrangement in Figure 4a–e is a combination of two *T*-droplets (at the center and upper right, circled in red) and five *M*-shells (2 + 5 combination), while Figure 4f shows a *T*-droplet surrounded by six *M*-shells (1 + 6 combination). In the 2 + 5 combination, the cross-communications occur between three combinations of various spherical structures, namely two *M*-shells, two *T*-droplets of same pitch and helicity handedness, and *M*-shells and *T*-droplets with opposite helicity handedness and different pitches.

Similar to the microshells discussed above, two CLC microdroplets with the same pitch and helicity handedness could form lateral communication, as shown in Figure 4g. It is worth mentioning that the NA of our objective was 0.25, thus the reflected light could be observed not only in a precisely vertical direction. As a matter of fact, asymmetric reflected path was allowed in a small range of incident and reflected angles [18]. Therefore, the reflections could be established as long as the condition *λ* = *n*_1_*p*_1_cos*θ*_1_ = *n*_2_*p*_2_cos*θ*_2_ was satisfied, as in the examples shown in Figure 4h,i. In Figure 4a–f, we could clearly identify the reflected spots with different pitches experimentally. As the temperature gradually rose, the pitch of *T*-droplets decreased and the wavelength of all the reflected spots blueshifted until the reflected light reached the invisible ultraviolet region. It was confirmed from Figure 4a,d that the cross-communication between two CLC spherical structures with opposite helicity handedness could still exist, although it was much weaker than that with the same helicity handedness. This agrees well with previous theoretical analysis and experimental results showing that the reflection should involve more complex polarization modes when the incident angle is not equal to zero [17,22,23]. This finding provides a possible way to control the reflected intensity at the same distance by changing the helicity handedness. 

### 3.3. Magnetic Control of Macroscopical Arrays for Secure Authentication

**Figure 5:**
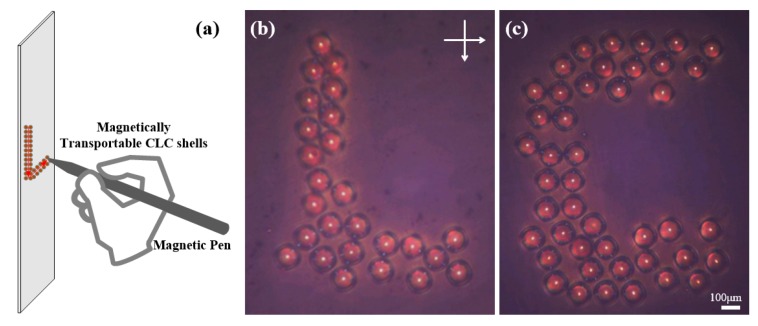
(**a**) Schematic diagram of controlling microshells by a magnetic pen; (**b**,**c**) POM images of the microshells arranged into English letters “L” and “C”.

Currently, spherical structures arranged in designed arrays are of particular interest for their promising applications in anti-forgery patterning [20]. Usually, these arrays are formed by depositing particles in pre-defined trenches, holes, or other templates fabricated via mechanical rubbing or photolithography [20,24]. Herein, we proposed a simple way to arrange spherical CLC structures into more complex patterns by taking advantage of their magnetic transportability. As shown in Figure 5a, we sealed a suitable number of *M*-shells in a rectangle glass cell and used a pen with a magnetic tip to manipulate them into uppercase English letters “L” and “C”, which were the initial characters of the words “Liquid” and “Crystal”. These microshells were positioned and arranged into the designed geometric figures, as exhibited in Figure 5b–c. In this manner, we can expect that more intricate patterns could be realized if the magnetic field is controlled precisely. Furthermore, the arrays with designed patterns arranged in this manner are reconfigurable in comparison to the aforementioned template-based methods. If magnetically transportable microshells with different helicity handedness and thermosensitivities are mixed to generate arrays, the spatial distribution of reflection spots with varying colors and intensities would respond to external stimuli, e.g., temperature and light, etc. Such dynamic changes, which are believed to be difficult to forge, can provide good photonic application prospects toward security authentication. Nevertheless, the temporal and mechanical stabilities of photonic cross-communication patterns are crucial for concrete applications, as we discuss above that the thermal agitation of surrounding fluids would possibly disturb the arrangement of shells in the absence of an external magnetic field. It was reported that the full photonic properties of spherical CLCs prepared with a reactive mesogen mixture can still be maintained after the extraction of a nonreactive chiral dopant [25]. Driven mainly by the surface and the interfacial tensions, these solidified CLC microspheres can interconnect with each other and sink into the polymer films they are deposited on after suitable vapor annealing processes [26]. This approach to improve stabilities can be applied in many CLC application fields that were restricted by LCs’ unstable fluidic state. 

## 4. Conclusions

In conclusion, we used microfluidic technology to incorporate water-soluble magnetofluid containing Fe_3_O_4_ nanoparticles into the inner aqueous core of CLC shells, responsible for the non-invasive transportable capability. The reflection interactions between neighboring spherical CLC Bragg reflectors were identified by varying the mutual distance in a group of magnetically transportable and unmovable microshells under a magnetic field. The temperature-dependent tunability of optical reflection patterns was investigated in close-packed hexagonal arrangements of seven CLC droplets and shells with different pitches and inverse helicity handedness. Moreover, we demonstrated that the magnetic field-assisted assembly of microshells arranged into arrays with geometric figures of uppercase English letters “L” and “C” can be successfully achieved for security authentication.
